# Shape-Persistent Tetraphenylethylene Macrocycle: Highly Efficient Synthesis and Circularly Polarized Luminescence

**DOI:** 10.3390/ma18010200

**Published:** 2025-01-05

**Authors:** Peixin Liu, Yuexuan Zheng, Zejiang Liu, Zhiyao Yang, Ziying Lu, Xiongrui Ai, Zecong Ye, Cheng Yang, Xiaowei Li, Lihua Yuan

**Affiliations:** 1 College of Chemistry, Sichuan University, Chengdu 610064, China; 2 School of Chemical Engineering and Light Industry, Guangdong University of Technology, Guangzhou 510006, China

**Keywords:** hydrogen-bonded macrocycle, aggregation-induced emission, tetraphenylethylene, self-assembly, circularly polarized luminescence

## Abstract

Circularly polarized luminescence (CPL) is an emerging field with significant applications in molecular electronics, optical materials, and chiroptical sensing. Achieving efficient CPL emission in organic systems remains a major challenge, particularly in the development of materials with high fluorescence quantum yields (Φ_F_) and large luminescence dissymmetry factors (g_lum_). Herein, we report the efficient synthesis of shape-persistent tetraphenylethylene macrocycles and investigate its potential as a CPL material. Chiral side chains were introduced to induce chiroptical properties. The macrocycles and their properties were characterized using NMR, MALDI-TOF MS, FT-IR, TGA, DSC, UV-Vis spectroscopy, SEM, fluorescence spectroscopy, ECD, and CPL. A significant fluorescence enhancement was observed upon aggregation, demonstrating a typical aggregation-induced emission (AIE) behavior. Moreover, one of the macrocycles in the solid state displayed distinct CPL emission with a high g_lum_ of 2 × 10^−2^ and a Φ_F_ value reaching 60%, and exhibited aggregation-induced circularly polarized luminescence (AICPL). These findings highlight the advantage of using a macrocycle with a noncollapsible backbone for the design of organic systems with CPL property, offering promising applications in chiroptical materials.

## 1. Introduction

Chirality is a phenomenon widely observed in nature, playing a crucial role in numerous life processes [1]. For instance, diverse proteins essential to all living organisms are constructed from chiral amino acids [2]. Beyond its occurrence in physical substances, chirality also exists in electromagnetic waves [3]. A notable example is circularly polarized light, which can be generated by passing natural light sequentially through a polarizer and a quarter-wave plate, resulting in light with an electromagnetic field that rotates helically around the optical axis. Circularly polarized light represents a significant form of asymmetric light. To date, circularly polarized light has proven indispensable in fields such as 3D displays [4], quantum computing [5], optical sensors [6], and asymmetric synthesis [7]. However, the process of obtaining circularly polarized light through optical filtering is often accompanied by significant energy loss, motivating extensive research into the direct generation of circularly polarized light from the origin [3].

Circularly polarized luminescence (CPL) is a way of generating circularly polarized light through fluorescent molecules, which has garnered considerable research interest to date [8,9,10,11,12]. Achieving efficient CPL requires that fluorescent molecules simultaneously exhibit high fluorescence quantum yield (Φ_F_) and a large luminescence dissymmetry factor (g_lum_) [8]. Self-assembly provides an effective and convenient strategy to enhance the luminescence dissymmetry factor of CPL-active materials, as it can amplify the g_lum_ value by 1–2 orders of magnitude, greatly improving the asymmetry of CPL emission [13]. However, during the aggregation process, conventional fluorescent molecules often face the challenge of aggregation-caused quenching (ACQ), which decreases the Φ_F_ while increasing the g_lum_, or even leads to a complete loss of fluorescence in aggregate state [14]. To address this contradiction, the key lies in the use of aggregation-induced emission (AIE) molecules, which exhibit properties opposite to those of ACQ molecules, as CPL emitters [15]. Unlike ACQ molecules, AIE molecules such as tetraphenylethylene (TPE) exhibit minimal fluorescence in the solution state but show enhanced emission in the aggregate state. This unique characteristic allows AIE molecules to improve both g_lum_ and Φ_F_ through self-assembly, thereby synergistically enhancing their overall CPL performance [13].

In recent years, the advancement of supramolecular chemistry has provided novel strategies for the design and modulation of CPL materials by introducing macrocyclic frameworks or helical foldamers into AIE emitters [9,10]. Not only is the cyclization itself a restriction on the conformation, but the motion of the TPE unit after cyclization is hindered as well, which, according to the restriction of intramolecular motions (RIM) mechanism, enhances luminescence by reducing non-radiative decay and raises the racemization energy barrier, facilitating the improvement of the g_lum_ value. [16]. In 2016, a BODIPY-based macrocycle with a figure-of-eight helicity was synthesized and was proven to be an efficient red-emitting CPL fluorophores with the g_lum_ factor reaching up to 9 × 10^−3^ [17]. In 2022, a triangular macrocycle containing chiral vertices revealed chiral self-assembly controlled by planar chirality, resulting in CPL emission [18]. In 2023, a chiral macrocycle constructed from substituted TPE and binaphthol exhibited CPL emission in the aggregate state, with a g_lum_ factor reaching 2 × 10^−3^ [19]. Despite these advancements, constructing macrocycles that simultaneously exhibit high g_lum_ and Φ_F_ remains a challenging task.

**Figure 1:**
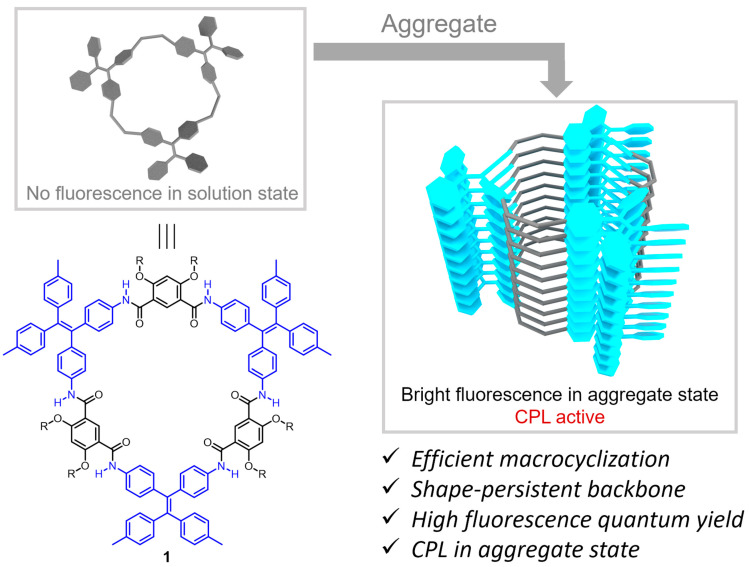
Molecular design of shape-persistent tetraphenylethylene macrocycle **1**.

Among various supramolecular cyclic systems, hydrogen-bonded aramide macrocycles [20] stand out for their shape-persistent structures, electron-rich cavities, and ease of derivatization, making them suitable for applications in recognition [21], liquid crystal materials [22], catalysis [23], and artificial molecular machines [24]. Given the success of reported examples in improving CPL performance with a marriage of TPE and cyclic structures, we envisioned that incorporation of TPE units into a hydrogen-bonded aromatic amide macrocycle might enable enhancement of the luminescence of TPE. The hydrogen-bonded aromatic amide structure is particularly well suited for this purpose because it provides a highly rigid framework that not only stabilizes the conformation of the macrocycle but also minimizes non-radiative decay pathways through efficient restriction of intramolecular motion. Additionally, the electron-rich aromatic amide backbone facilitates stronger π-π interactions in the self-assembly process, further amplifying both AIE and CPL effects. Since the presence of intramolecular hydrogen bonds in the molecular backbone endows the macrocycle with shape-persistency, this design significantly restricts internal molecular motion. Inspired by previous research [25], we designed hydrogen-bonded aramide macrocycle 1 (Figure 1). Chiral side chains are tethered to the periphery of the macrocycle to produce chiroptical properties. Additionally, the presence of aromatic units partially rigidified by intramolecular hydrogen bonds offers shape-persistency of the molecular backbone, and helps facilitate self-assembly, which is expected to increase Φ_F_ and g_lum_ values. Such a design features high modifiability, allowing the incorporation of different side chains to fine-tune its properties. It is worth noting that the conformational rigidity of the TPE units is reinforced not only by strain caused by cyclization, but also by the restriction of intramolecular motion (RIM), owing to decreased flexibility of the local backbone comprising three hydrogen-bonded aromatic amide units. This favors the expression of distinct chiroptical properties upon the introduction of chirality into the ring. The efficient formation of the shape-persistent tetraphenylethylene macrocycle was achieved, and the AIE phenomenon was observed in the macrocycle. One of the macrocycles bearing chiral side chains exhibited CPL emission with a g_lum_ factor as high as 2 × 10^−2^.

## 2. Results and Discussion

### 2.1. Synthesis

**Scheme 1:**
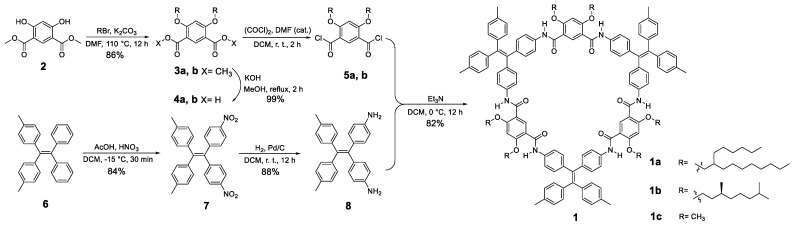
Synthetic route of shape-persistent tetraphenylethylene macrocycles **1a**–**1c**. Macrocycle **1c** is used for DFT computation.

The chemical structure and synthetic route of the shape-persistent tetraphenylethylene macrocycles are illustrated in Scheme 1. Starting from dimethyl ester **2** and the corresponding alkyl bromide, compound **3a** (or **3b**) was synthesized by nucleophilic substitution reaction to introduce branched side chains (for **3a**) and chiral side chains (for **3b**) in yields of ca. 86%. Hydrolysis of the diester under basic conditions afforded diacid compound **4a** (or **4b**) an almost quantitative yield. Acyl chloride **5a** (or **5b**) was prepared in situ from its corresponding acid by reaction with oxalyl chloride and used immediately for the subsequent coupling reaction. Compound **8** was synthesized by subjecting compound **6** to nitration, producing compound **7**, which was then followed by reduction [25]. Different from the reported procedure where the reduction was implemented with NH_2_-NH_2_, hydrogenation was employed here over a Pd/C catalyst, which achieved compound **8** in 88%. Finally, precursors **5a** (or **5b**) and **8** were efficiently condensed in DCM to form macrocycle **1** through an amide bond formation reaction. The high yield of macrocycles could be attributed to the spatial folding of the oligomeric intermediates as a result of the stabilizing intramolecular hydrogen bonds, as supported by computational results (*vide post*).

**Figure 2:**
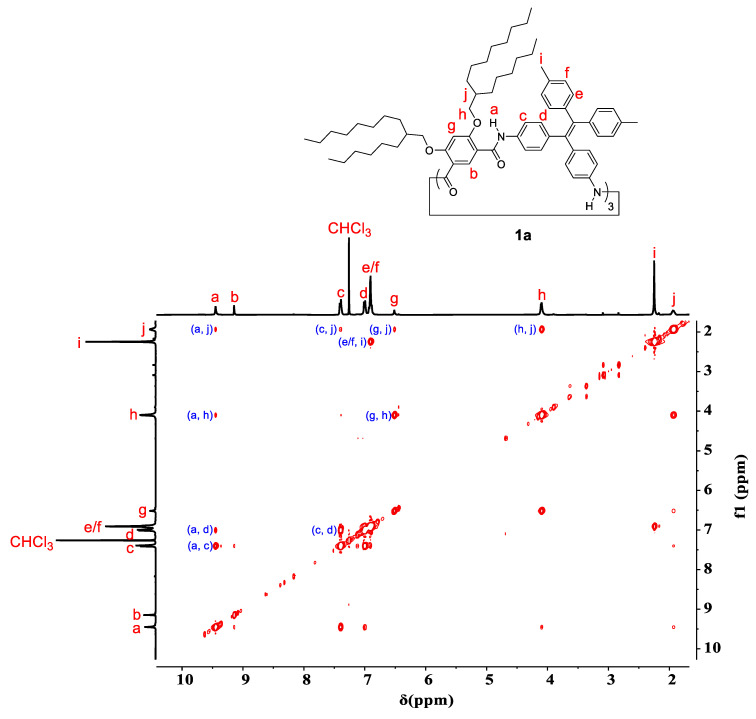
Expanded 2D-NOESY spectrum of compound **1a** (400 MHz, CDCl_3_, 298 K, mixing time = 0.4 s).

The structures of the intermediates and the macrocycles were confirmed by NMR, FT-IR, MALDI-TOF-MS, TGA, and DSC (Figures S1–S15). The ^1^H-NMR spectrum of compound **1a** (Figure S6) shows amide N-H proton signals in the low-field region, indicating the presence of amide linkage. The MALDI-TOF-MS data of **1a** and **1b** (Figures S8 and S9) further confirmed the molecular structure. To evidence the presence of intramolecular hydrogen bonds in the macrocycle, a 2D-NOESY experiment was carried out with compound **1a**. The results (Figure 2 and Figure S7) reveal a short spatial distance and coupling between the amide proton H^a^ and the protons H^h^ and H^j^ on the alkoxy side chains, supporting the conformation of macrocycle with amide oxygen oriented towards the cavity and the presence of intramolecular hydrogen bonds. The FT-IR spectra of compounds **1a** and **1b** (Figures S10 and S11) are nearly identical, indicating their structural consistency. The broad absorption band at 3400–3300 cm^−1^ corresponds to N–H stretching vibrations of the amide group, also indicating intramolecular hydrogen bonding. In the 3000–2800 cm^−1^ region, narrow bands correspond to C-H stretching vibrations of aromatic and aliphatic groups, while a strong absorption band at ~1700 cm^−1^ is attributed to C=O stretching vibrations in the amide group. These spectral features of the macrocycles collectively confirm its structure. Thermogravimetric analysis and differential scanning calorimetry (Figures S12–S15) collectively confirm the good thermal stability of both macrocycles.

### 2.2. Molecular Conformation

After numerous attempts, we were unable to obtain a single crystal structure of macrocycle **1**, possibly due to the strong tendency of the macrocycles to molecular aggregation and the presence of bulky side chains that hinder the orderly packing of the macrocycles. To gain insights into the molecular conformation of the macrocycle, we conducted a detailed analysis using theoretical calculations. The conformation of macrocycle **1** was optimized and analyzed using density functional theory (DFT) at the B3LYP/6-31G(d) level.

**Figure 3:**
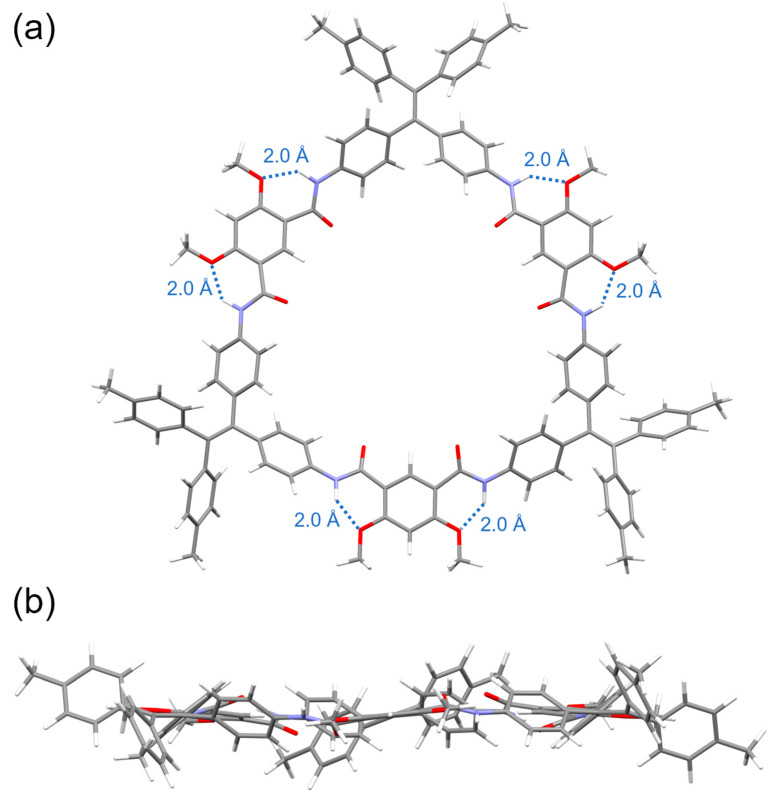
Top view (**a**) and side view (**b**) of optimized geometry of macrocycle **1c** at the B3LYP/6-31G(d) level (gray = C, white = H, red = O, and blue = N).

The computational results revealed that the macrocycle adopted a stable near-planar ring structure stabilized by intramolecular hydrogen bonds (~2.0 Å) with a cavity in size of approximately 13.0 Å in diameter (Figure 3a). Notably, multiple hydrogen bonds were observed between the N-H hydrogen atoms of the amide groups and the oxygen atoms of the alkoxy side chains. These hydrogen bonds not only play a crucial role in pre-organizing the shape-persistent backbone of aromatic amide fragments in a curvature fashion during the cyclization process, leading to the efficient formation of shape-persistent macrocycle rather than the production of overshooting products such as helical oligomers or polymers [26], but also enhance rigidity and overall stability of the macrocycle. The intramolecular hydrogen bonds maintain the near-planar conformation of the macrocycle (Figure 3b).

### 2.3. Photophysical Property

**Figure 4:**
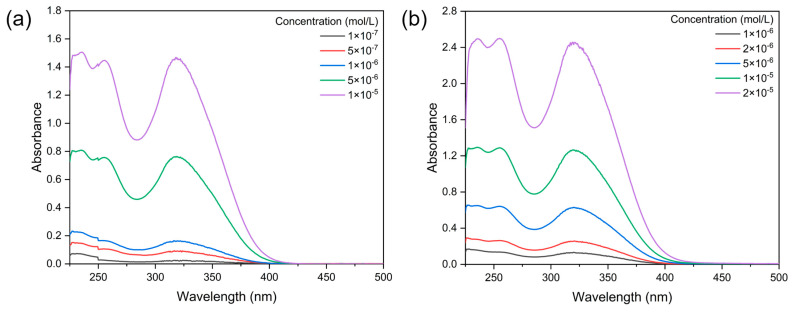
The absorption spectra of (**a**) **1a** in DCM, and (**b**) **1b** in DCM.

Macrocycles **1a** and **1b** exhibit strong UV absorption in DCM (Figure 4), displaying characteristic absorption peaks of TPE derivatives. Both macrocycle **1a** and **1b** present three UV absorption peaks at 235 nm, 255 nm, and 320 nm. Although there are slight differences in the maximum absorption wavelengths, the overall shapes of the UV absorption spectra of macrocycle **1a** and **1b** are essentially identical, indicating a high degree of similarity in their conjugated systems.

**Figure 5:**
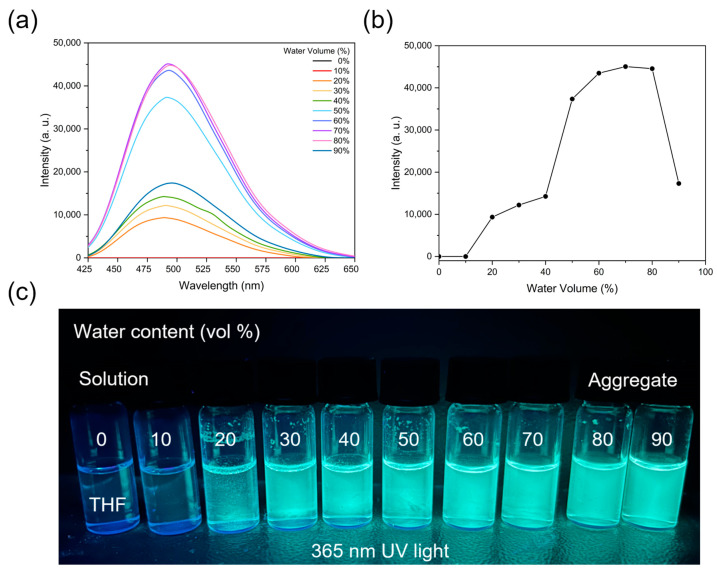
(**a**) Change in the fluorescence spectra of **1a** in H_2_O/THF mixed solvent with water fraction ([**1a**] = 1.0 × 10^−4^ M, λ_ex_ = 395 nm, ex/em slit widths = 1/1 nm). (**b**) The curve of fluorescence spectra intensity of **1a** in H_2_O/THF mixed solvent at 495 nm vs. water volume percent ([**1a**] = 1.0 × 10^−4^ M, λ_ex_ = 395 nm, ex/em slit widths = 1/1 nm). (**c**) Photograph of **1a** in THF with different water fractions under 365 nm UV light.

To examine the AIE properties of the shape-persistent tetraphenylethylene macrocycle, **1a** was fully soluble in tetrahydrofuran (THF) and thus opted for fluorescence experiments. Fluorescence spectra were measured under various mixed solvent conditions by adjusting the THF-to-water ratio. In pure THF, **1a** exhibits almost no fluorescence emission, indicating a non-emissive state in solution. This phenomenon can be attributed to the efficient dissipation of the excited-state energy through non-radiative transitions in the monomeric state. As water, a poor solvent was gradually introduced, a significant fluorescence enhancement was observed (Figure 5a,b). At a water content of 80%, the fluorescence intensity reached its maximum, exceeding that in pure THF by over 10,000 times. The fluorescence spectra revealed a marked increase in emission intensity as molecular aggregation progressed. This behavior can be attributed to the RIM mechanism of AIE, where the restriction of intramolecular motion in the aggregate state suppresses non-radiative decay pathways, effectively channeling the excited-state energy into radiative transitions and thereby significantly enhancing fluorescence emission. A red shift in the maximum emission peak was also observed with increasing water content, which can be attributed to the formation of J-aggregates. [27] Under 365 nm UV light, the macrocycle exhibited a cyan-green fluorescence (Figure 5c). These results indicate that the shape-persistent tetraphenylethylene macrocycle is a typical AIE-active compound, exhibiting a pronounced fluorescence enhancement with aggregation. To further investigate the fluorescence properties of the macrocycle, the fluorescence quantum yield and transient fluorescence decay spectra of the macrocycle **1a** and **1b** were determined. The results showed (Figures S16–S19) that the fluorescence quantum yields (Φ_F_) of **1a** and **1b** were 60% and 41%, respectively, and the fluorescence lifetimes (τ) were 5.08 ns and 4.22 ns, respectively.

### 2.4. Chiral Optical Properties

**Figure 6:**
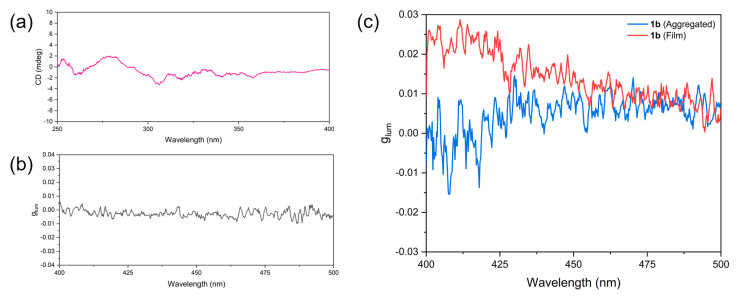
(**a**) ECD spectra of **1b** (1 × 10^−5^ M, DCM). (**b**) CPL result of **1b** (1 × 10^−5^ M, DCM) (**c**) CPL result of aggregated **1b** (1 × 10^−5^ M, DCM/Hexane = 1:9, λ_em_ = 340 nm). All the spectra were recorded at 298 K.

Typically, AIE compounds adopt a propeller-like structure, which naturally forms either a left-handed or right-handed helical conformation [8]. However, due to the lack of rotational barriers, these conformations can rapidly interconvert, resulting in a racemic state at the macroscopic level. By restricting all four sides of the TPE unit to prevent rapid interconversion between the two conformations, it is possible to isolate left-handed and right-handed enantiomers, each capable of exhibiting distinct electronic circular dichroism (ECD) and CPL signals [28]. The chiroptical properties of **1b** in DCM were first examined. At a concentration of 1 × 10^−5^ M, ECD and CPL measurements were conducted. As expected, no significant ECD or CPL signals were observed (Figure 6a,b). Next, after adding a poor solvent to induce aggregation, the chiroptical properties of the macrocycle in the aggregate state were investigated. CPL tests were then conducted in a nonpolar mixed solvent (DCM/Hexane, 1:9, *v*/*v*) and thin film on a quartz substrate. The results (Figure 6c) showed distinct asymmetric emissions in both aggregate states. This indicates that the macrocycle exhibited no significant chiroptical properties in the non-aggregate state but demonstrated obvious CPL emission in the aggregate state, showcasing pronounced aggregation-induced circularly polarized luminescence (AICPL) characteristics [8]. The g_lum_ value was found to reach 2 × 10^−2^, which is considered a notable improvement compared to the majority of previously reported organic macrocycle-based CPL materials [9].

### 2.5. Morphology of Aggregate State

**Figure 7:**
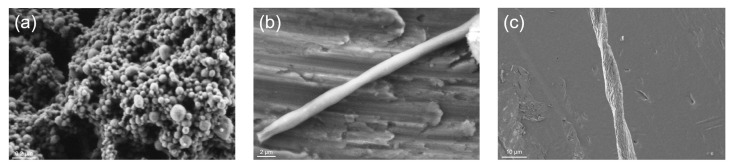
SEM images of **1b** (**a**) assembled in a mixed solvent of DCM and MeOH (1:9, *v*/*v*); (**b**) assembled in p-xylene/hexane (1:9, *v*/*v*); (**c**) assembled in p-xylene/hexane (1:9, *v*/*v*), amplified region showing one helical bundle.

The CPL amplification effect in the aggregate state is typically related to the self-assembly. To investigate the self-assembly behavior of the shape-persistent tetraphenylethylene macrocycle in different solvents, a scanning electron microscopy (SEM) experiment was conducted with **1b** in polar solvent (DCM/MeOH, 1:9, *v*/*v*) and non-polar solvent (p-xylene/hexane, 1:9, *v*/*v*). In the polar solvent (Figure 7a), the macrocycle tends to form dense spherical aggregates. In sharp contrast, in non-polar solvents (Figure 7b,c), the macrocycle exhibits distinct bundled structures in which clear helical chiral features were observed. The large difference in solvents of varying polarity indicates that the polarity of the solvent significantly influences the self-assembly morphology of the macrocycle. In a non-polar environment, due to the poor solubility effect, the π-π interactions between the molecules are significantly enhanced, resulting in more orderly stacking of the molecules into helical bundled structures. This ordered supramolecular structure facilitates the effective amplification of chiral signals.

## 3. Materials and Methods

### 3.1. Materials and Reagents

All chemicals used in this study were purchased from commercial sources and used without further purification unless specified otherwise. Reactions were performed in oven-dried glassware under nitrogen or ambient atmosphere, using Teflon-coated magnetic stir bars. Solvents were dried and distilled according to standard procedures. Reaction progress was monitored via thin-layer chromatography (TLC). Work-up and purification steps utilized reagent-grade solvents under ambient conditions. Column chromatography was carried out on silica gel (100–200 mesh and 300–400 mesh). NMR solvents were procured from Energy Chemical (Shanghai, China).

### 3.2. Experimental Methods

NMR data were measured and collected using a Bruker DRX 400 spectrometer (Karlsruhe, Germany). UV-Vis spectra were obtained using a SHIMADZU UV-2600 spectrophotometer (Kyoto, Japan). Fluorescence spectra were measured using an FL970 instrument from Shanghai Tianmei Scientific Instrument Co., Ltd (Shanghai, China). Circular dichroism spectra were recorded using a JASCO J-1500-150ST spectrometer (Tokyo, Japan). Circularly polarized luminescence spectra were collected with an Olis CPL Solo spectrometer (Athens, GA, USA). MALDI-TOF-MS data were acquired using a SHIMADZU AXIMA performance instrument (Kyoto, Japan). SEM images were obtained using a Zeiss Sigma 300 scanning electron microscope (Oberkochen, Germany).

### 3.3. DFT Calculations

Geometrical optimizations were conducted using DFT calculations within the Gaussian 09 program package [29].

### 3.4. Synthesis of Shape-Persistent Tetraphenylethylene Macrocycle

The synthesis route of the shape-persistent tetraphenylethylene macrocycle is shown in Scheme 1. Compounds **2** and the brominated chiral side chain derivative were synthesized following literature methods [30,31].

#### 3.4.1. Synthesis of Compound **3**

To a 500 mL single-neck round-bottom flask, compound **2** (10 mmol, 1.0 equiv.) and K_2_CO_3_ (50 mmol, 5.0 equiv.) were added in DMF (100 mL). The mixture was heated to 80 °C and stirred for 30 min. The bromo compound (22 mmol, 2.2 equiv.) was added to the mixture above, and the temperature was raised to 110 °C, followed by stirring at this temperature for 12 h. The reaction progress was monitored by TLC (eluent: DCM). Once the reactant was fully consumed, the reaction stopped. The reaction mixture was filtered, and the filtrate was concentrated under reduced pressure to remove most of the solvent. The residue was extracted with EA (100 mL) and water (100 mL), and the aqueous phase was extracted twice with EA (2 × 50 mL). The organic layers were combined and washed sequentially with 1 M KOH aqueous solution (150 mL), distilled water (150 mL), and saturated brine (150 mL). The organic layer was dried over anhydrous Na_2_SO_4_, filtered, and concentrated under reduced pressure to obtain the crude product of compound **3**. Compound **3** was purified by silica gel column chromatography (eluent: DCM).

#### 3.4.2. Synthesis of Compound **4**

To a 250 mL single-neck round-bottom flask, compound **3** (5 mmol, 1.0 equiv.) was added and dissolved in anhydrous methanol (50 mL). A saturated aqueous solution of KOH (25 mmol, 5.0 equiv.) was added to the flask, and the mixture was heated under reflux with an oil bath for 2 h. The reaction progress was monitored by TLC (eluent: DCM/MeOH, 50:1, *v*/*v*). The reaction mixture was allowed to cool down to room temperature and neutralized with dilute hydrochloric acid to pH 1–2. The solvent was removed under reduced pressure. The residue was extracted with DCM (50 mL) and water (50 mL), and the aqueous phase was extracted twice with DCM (2 × 50 mL). The organic layers were combined and washed sequentially with distilled water (150 mL) and saturated brine (150 mL). The organic layer was dried over anhydrous Na_2_SO_4_, filtered, and concentrated under reduced pressure to afford compound **4** (99% yield).

**4a** ^1^H NMR (400 MHz, CDCl_3_, 298 K) δ 10.22 (s, 2H), 8.98 (s, 1H), 6.56 (s, 1H), 4.16 (d, *J* = 5.2 Hz, 4H), 1.92 (m, 2H), 1.46 (m, 12H), 1.32–1.26 (m, 48H).

**4b** ^1^H NMR (400 MHz, DMSO-*d_6_*, 298 K) δ 12.30 (s, 2H), 8.17 (s, 1H), 6.70 (s, 1H), 4.17 (d, *J* = 5.7 Hz, 4H), 1.75 (m, 4H), 1.60–1.43 (m, 4H), 1.30 (m, 4H), 1.24 (m, 2H), 1.14 (m, 6H), 0.91 (d, *J* = 6.5 Hz, 6H), 0.84 (d, *J* = 6.6 Hz, 12H).

#### 3.4.3. Synthesis of Compound **5**

To a 100 mL round-bottom flask, compound **4** (1 mmol, 1.0 equiv.) was added and dissolved in anhydrous DCM (10 mL). Oxalyl chloride (5 mmol, 5.0 equiv.) and 10 μL of DMF were added to the flask, and the mixture was stirred at room temperature for 30 min. The reaction progress was monitored by TLC (eluent: DCM). When the reaction was complete, the mixture was concentrated under reduced pressure, and subjected to vacuum drying. The resulting product **5** was dissolved in anhydrous DCM, transferred to a constant-pressure dropping funnel, and directly used in the next reaction step.

#### 3.4.4. Synthesis of Compound **7**

To a 250 mL two-neck round-bottom flask, 14.4 g (40.0 mmol, 1.0 equiv.) of compound **6** was added and dissolved in 150 mL of DCM. A constant pressure dropping funnel was attached to the flask. To the reaction mixture, 11.4 mL (200 mmol, 5.0 equiv.) of glacial acetic acid was added, and the mixture was stirred at −15 °C for 10 min. Then, 15.8 mL of concentrated nitric acid (67%, 240 mmol, 6.0 equiv.) was added to the dropping funnel and slowly added dropwise to the reaction mixture while maintaining the temperature at −15 °C. The mixture was stirred for an additional 20 min at −15 °C. Next, 150 mL of distilled water was placed in a 500 mL beaker and cooled to 0 °C. The reaction mixture was then poured into the ice-cold water, and the mixture was extracted with DCM (3 × 50 mL). The organic layer was washed twice with 150 mL of distilled water and once with 150 mL of saturated brine. The organic phase was dried over anhydrous Na_2_SO_4_, filtered, and the solvent was removed under reduced pressure to afford the crude product of compound **7**. To the crude product, a small amount of DCM was added to dissolve it completely. Methanol was then poured into the solution until a large amount of solid precipitated. The precipitate was filtered, and the filter cake was washed several times with methanol to yield the pure compound **7** as a yellow solid (86% yield).

^1^H NMR (400 MHz, CDCl_3_, 298 K) δ 7.99 (d, *J* = 8.9 Hz, 2H), 7.15 (d, *J* = 8.9 Hz, 2H), 6.95 (d, *J* = 7.7 Hz, 2H), 6.88 (d, *J* = 8.1 Hz, 2H), 2.29 (s, 3H).

#### 3.4.5. Synthesis of Compound **8**

To a 250 mL round-bottom flask, 2.00 g (4.44 mmol) of compound **7** was added and dissolved in 50 mL of DCM and 5 mL of ethanol. Then, 350 mg (18% wt.) of Pd/C was added. The reaction was stirred under a hydrogen atmosphere in the dark for 12 h. The reaction progress was monitored by TLC (eluent: DCM), and the reaction was stopped once the reactant was completely consumed. The mixture was filtered, and the solvent was removed under reduced pressure to obtain the crude product of compound **8**. Recrystallization from DCM/methanol afforded the pure compound **8** as a yellow solid (88% yield).

^1^H NMR (400 MHz, CDCl_3_, 298 K) **δ** 6.89 (s, 4H), 6.81 (d, *J* = 8.0 Hz, 2H), 6.50–6.35 (d, *J* = 8.0 Hz, 2H), 3.55 (s, 2H), 2.25 (s, 3H).

#### 3.4.6. Synthesis of Compound **1**

To a 500 mL three-neck flask, compound **8** (1 mmol, 1.0 equiv.) was added and dissolved in 100 mL of anhydrous DCM. Et_3_N (5 mmol, 5.0 equiv.) was then added, and a pressure-equalizing dropping funnel was installed. The reaction system was purged with argon and stirred at room temperature for 1 h, then cooled to 0 °C. Compound **5** was dissolved in 100 mL of DCM and transferred to the dropping funnel, then slowly added dropwise to the three-neck flask. After the addition was complete, the reaction mixture was stirred at 0 °C for 2 h, followed by stirring at room temperature for 12 h.

Acetyl chloride (50 μL) was added to the reaction mixture and stirred at room temperature for 1 h, followed by the addition of methanol (200 μL), and stirring continued for another hour. The reaction mixture was extracted with KOH aqueous solution (150 mL), and the aqueous phase was extracted twice with DCM (2 × 50 mL). The organic phases were combined and washed once with KOH aqueous solution (150 mL) and once with saturated brine (150 mL). The organic layer was dried over anhydrous Na_2_SO_4_, filtered, and the solvent was removed under reduced pressure to yield the crude product of compound **1**. The crude product was recrystallized once from DCM/PE and once from DCM/EtOH. The solid was filtered and washed with PE, EtOH, and ether, respectively, to obtain the pure compound **1** (82% yield).

**1a** ^1^H NMR (400 MHz, CDCl_3_, 298K) **δ** 9.46 (s, 2H), 9.15 (d, *J* = 2.4 Hz, 1H), 7.40 (d, *J* = 8.0 Hz, 4H), 7.00 (d, *J* = 8.1 Hz, 4H), 6.91 (d, *J* = 2.9 Hz, 4H), 6.51 (s, 1H), 4.10 (d, *J* = 5.2 Hz, 4H), 2.25 (s, 6H), 1.52–1.47 (m, 10H), 1.35 (s, 12H), 1.29–1.22 (m, 24H), 0.86 (t, *J* = 3.3 Hz, 16H). MALDI-TOF-MS, m/z calcd for [C_204_H_276_N_6_O_12_+H]^+^ 3005.12, found 3005.22.

**1b** MALDI-TOF-MS, m/z calcd for [C_168_H_204_N_6_O_12_+H]^+^ 2500.52, found 2501.49.

## 4. Conclusions

By introducing TPE groups into hydrogen-bonded aromatic amide macrocycles, novel TPE-based aromatic amide macrocycles were successfully synthesized with high yields. The pre-organizing effect of hydrogen bonding ensured high yield and a shape-persistent planar structure, while the incorporation of TPE endowed the macrocycle with typical AIE properties. By introducing chiral side chains into the macrocycle, an aggregation-induced circularly polarized luminescence (AICPL) effect was achieved. The system demonstrated both a relatively high fluorescence quantum yield (Φ_F_ = 0.6) and g_lum_ value (0.02) among pure organic macrocyclic systems. This work not only offers a novel approach for organic macrocyclic CPL systems but also paves the way for the design of tunable organic CPL-emitting materials.

## Data Availability

The original contributions presented in the study are included in the article and supplementary material, further inquiries can be directed to the corresponding authors.

## Supplementary Materials

The following supporting information can be downloaded at https://www.mdpi.com/article/10.3390/ma18010200/s1, Figure S1. ^1^H NMR spectrum of compound **6**, Figure S2. ^1^H NMR spectrum of compound **7**, Figure S3. ^1^H NMR spectrum of compound **8**, Figure S4. ^1^H NMR spectrum of compound **4a**, Figure S5. ^1^H NMR spectrum of compound **4b**, Figure S6. ^1^H NMR spectrum of compound **1a**, Figure S7. 2D-NOESY spectrum of compound **1a**, Figure S8. MALDI-TOF-MS spectrum of compound **1a**, Figure S9. MALDI-TOF-MS spectrum of compound **1b**, Figure S10. FT-IR spectrum of compound **1a**, Figure S11. FT-IR spectrum of compound **1b**, Figure S12. TGA and DTG diagrams of compound **1a**, Figure S13. TGA and DTG diagrams of compound **1b**, Figure S14. DSC diagrams of compound **1a**, Figure S15. DSC diagrams of compound **1b**, Figure S16. Quantum yield result of compound **1a**, Figure S17. Quantum yield result of compound **1b**, Figure S18. Transient fluorescence decay spectra of compound **1a**, and Figure S19. Transient fluorescence decay spectra of compound **1b**.
