# Supplementary material for: Shape-Persistent Tetraphenylethylene Macrocycle: Highly Efficient Synthesis and Circularly Polarized Luminescence
Source: Materials (Basel). 2025 Jan 5;18(1):200. doi: 10.3390/ma18010200 (PMC11722041; doi:10.3390/ma18010200)
Supplement: Supplementary file 1 [file materials-18-00200-s001.zip › materials-3389993-supplementary.pdf]

Supporting Information

Shape-persistent Tetraphenylethylene Macrocycle: Highly Efficient Synthesis and Circularly Polarized Luminescence

Peixin Liu [1], Yuexuan Zheng [1], Zejiang Liu [1], Zhiyao Yang [1], Ziying Lu [2], Xiongrui Ai [2], Zecong Ye [2], Cheng Yang [1], Xiaowei Li [1,*], and Lihua Yuan [1,*]

[1] College of Chemistry, Sichuan University, Chengdu 610064, Sichuan, China
[2] School of Chemical Engineering and Light Industry, Guangdong University of Technology, Guangzhou 510006, Guangdong, China
* Correspondence: lixw@scu.edu.cn (X. L.); lhyuan@scu.edu.cn (L. Y.)

Contents

1. NMR Spectra S3

2. MALDI-TOF-MS Spectra S7

3. FT-IR Spectra S8

4. Thermogravimetric Analysis S9

5. Differential Scanning Calorimetry Results S10

6. Quantum Yield Results S11

7. Transient Fluorescence Decay Spectra S12

8. DFT Calculation Result S13

1. NMR Spectra

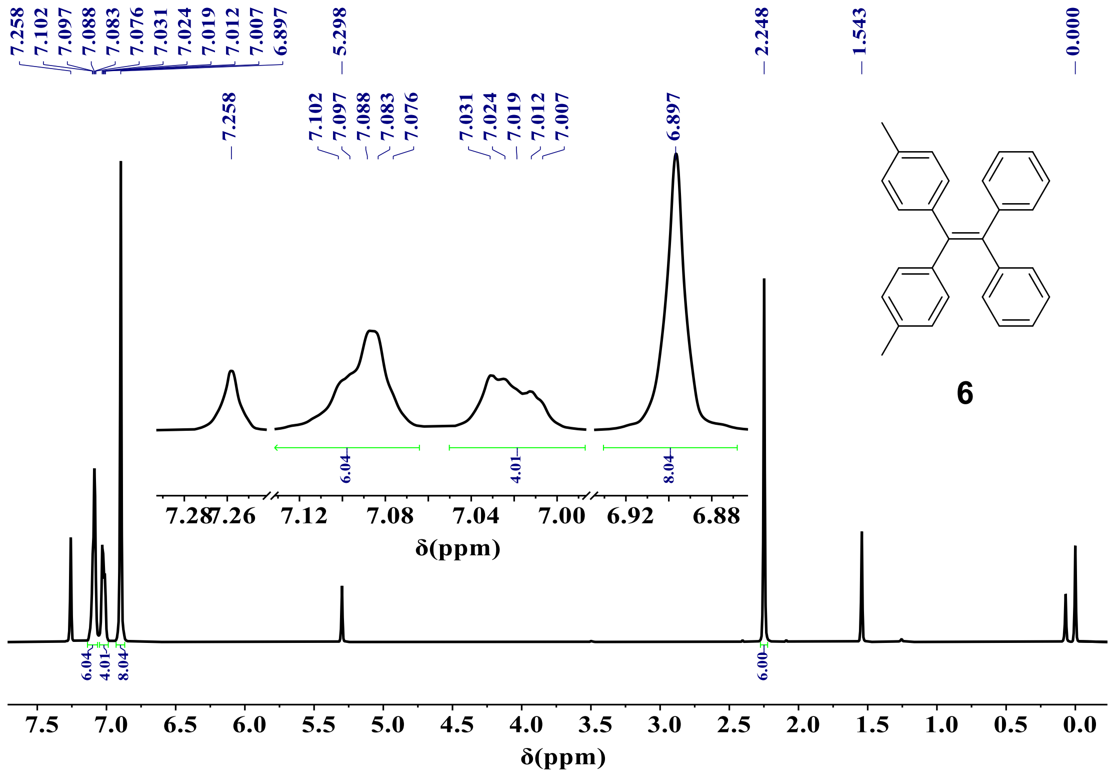

Figure S1. ^{1}H NMR spectrum of compound **6** (400 MHz, $CDCl_3$, 298 K)

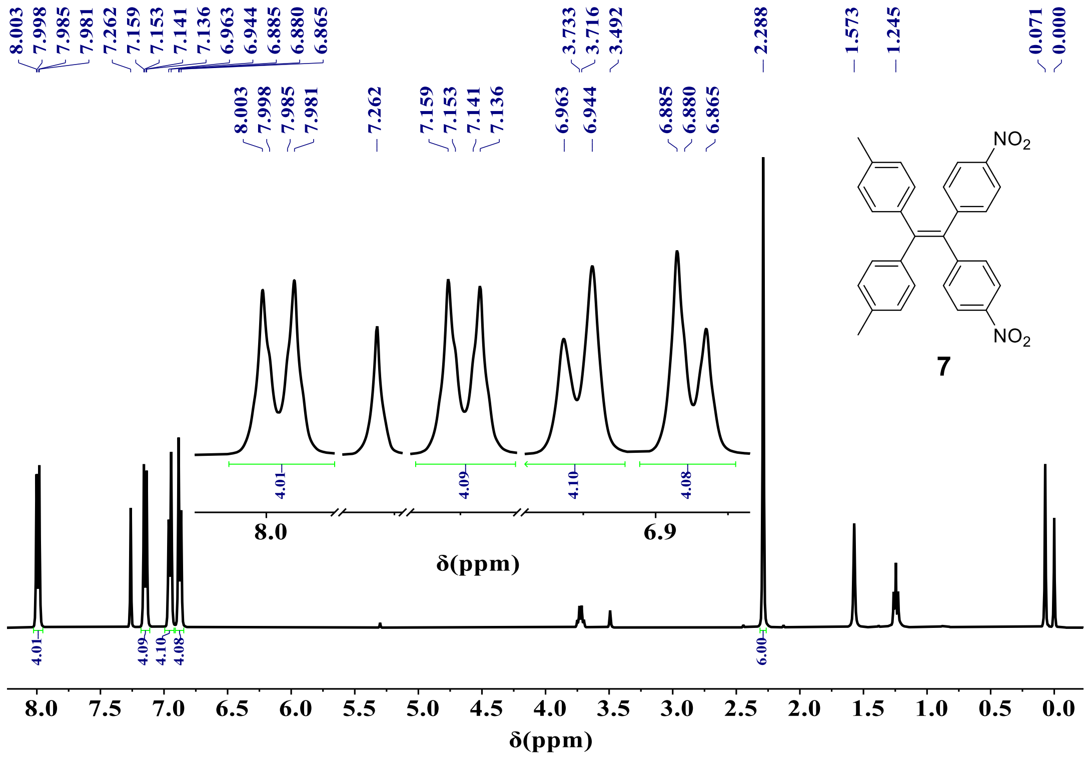

Figure S2. ^{1}H NMR spectrum of compound **7** (400 MHz, $CDCl_3$, 298 K)

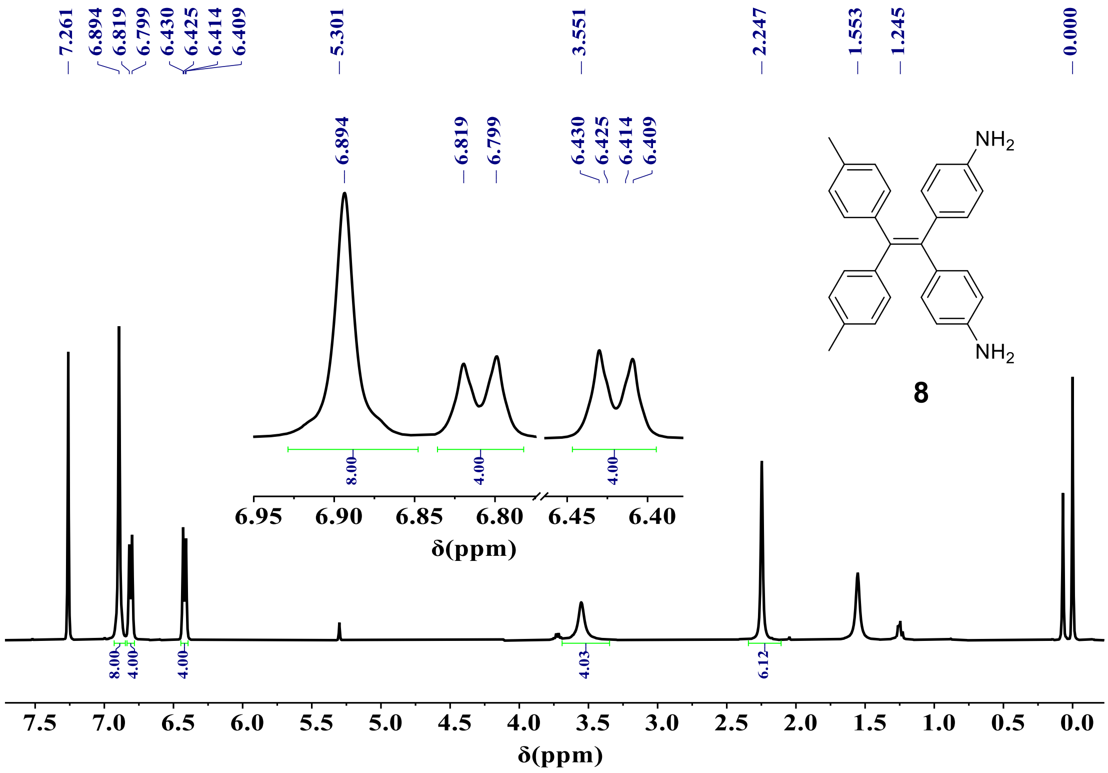

Figure S3. ^{1}H NMR spectrum of compound **8** (400 MHz, $CDCl_3$, 298 K)

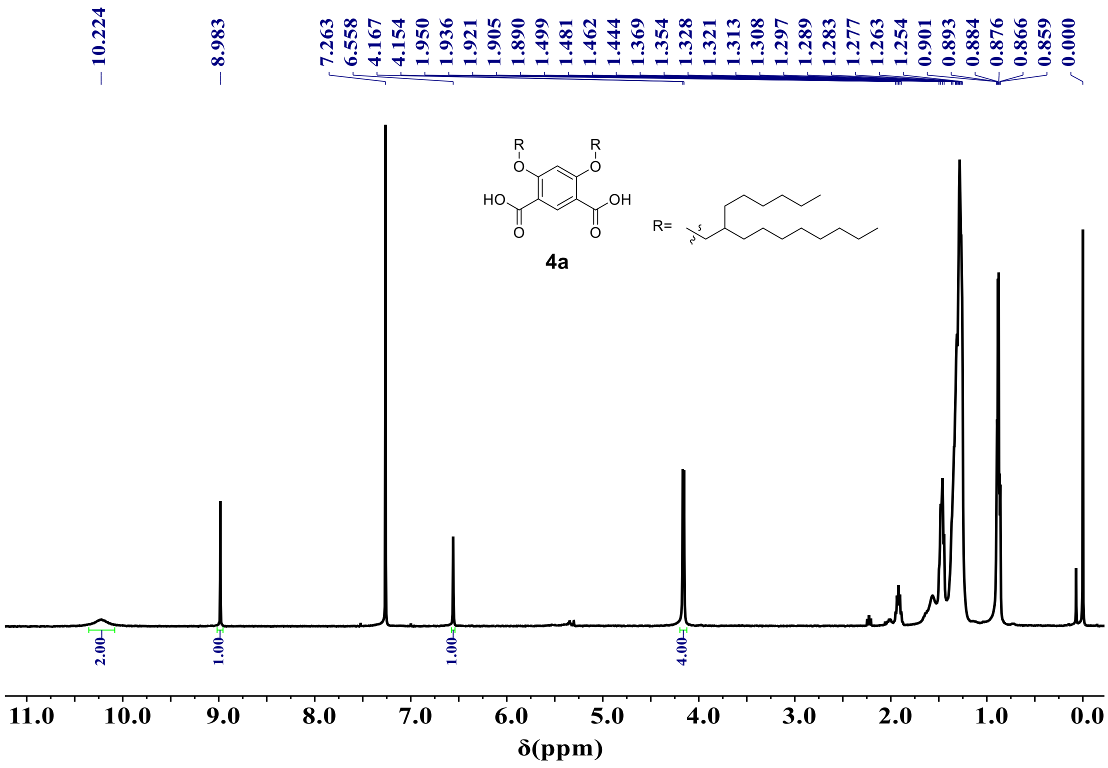

Figure S4. ^{1}H NMR spectrum of compound **4a** (400 MHz, $CDCl_3$, 298 K)

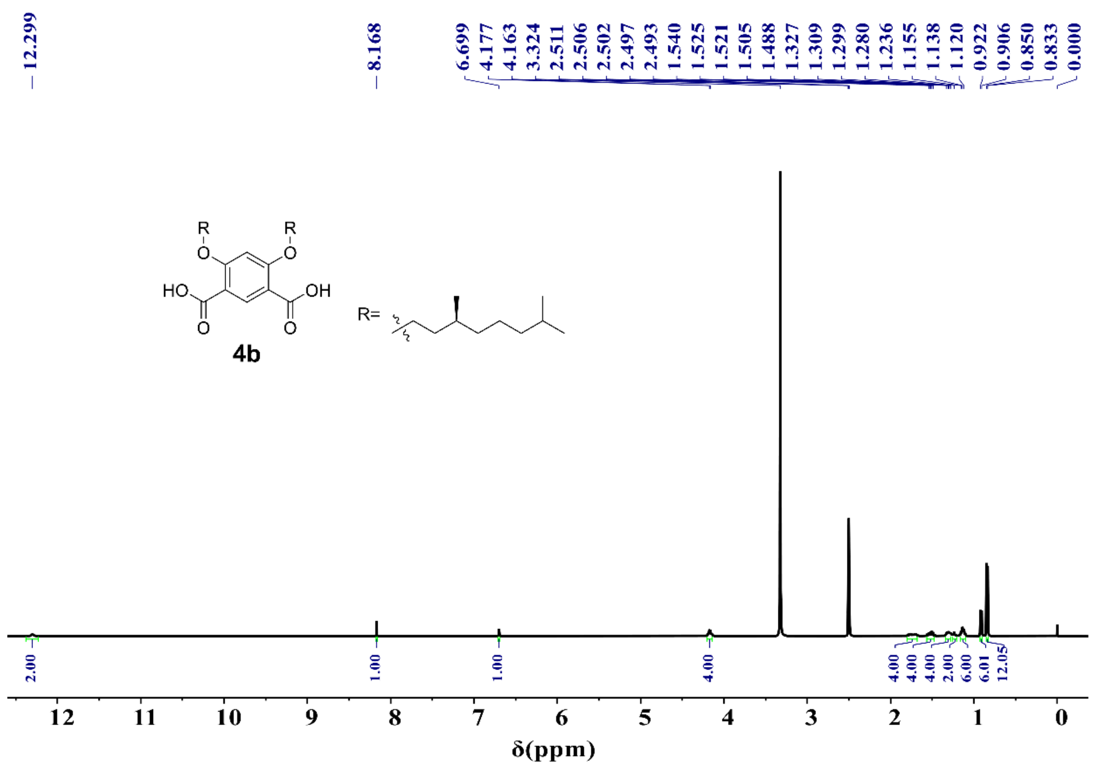

Figure S5. ^{1}H NMR spectrum of compound **4b** (400 MHz, DMSO-*d6*, 298 K)

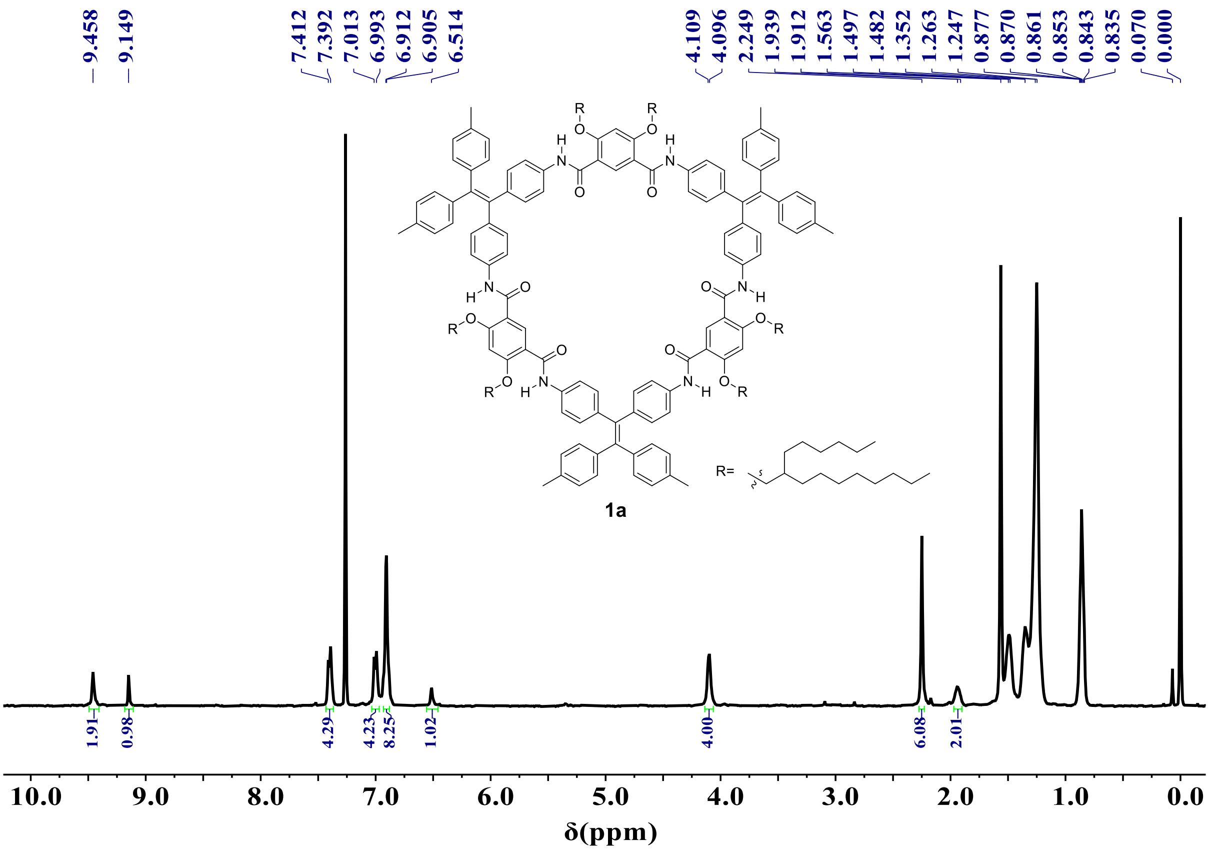

Figure S6. ^{1}H NMR spectrum of compound **1a** (400 MHz, $CDCl_3$, 298 K)

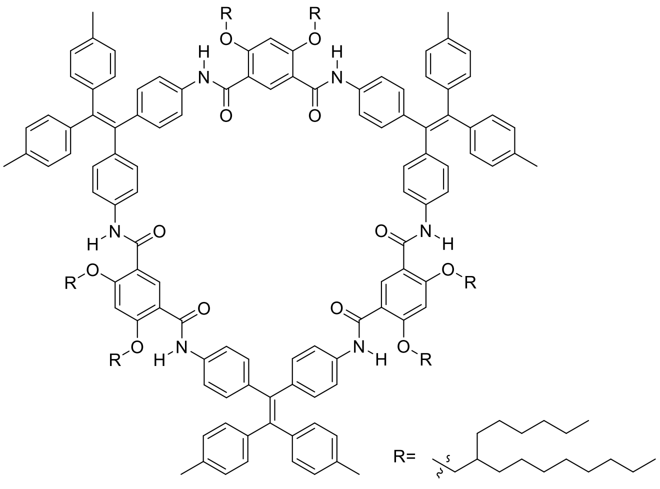

1a

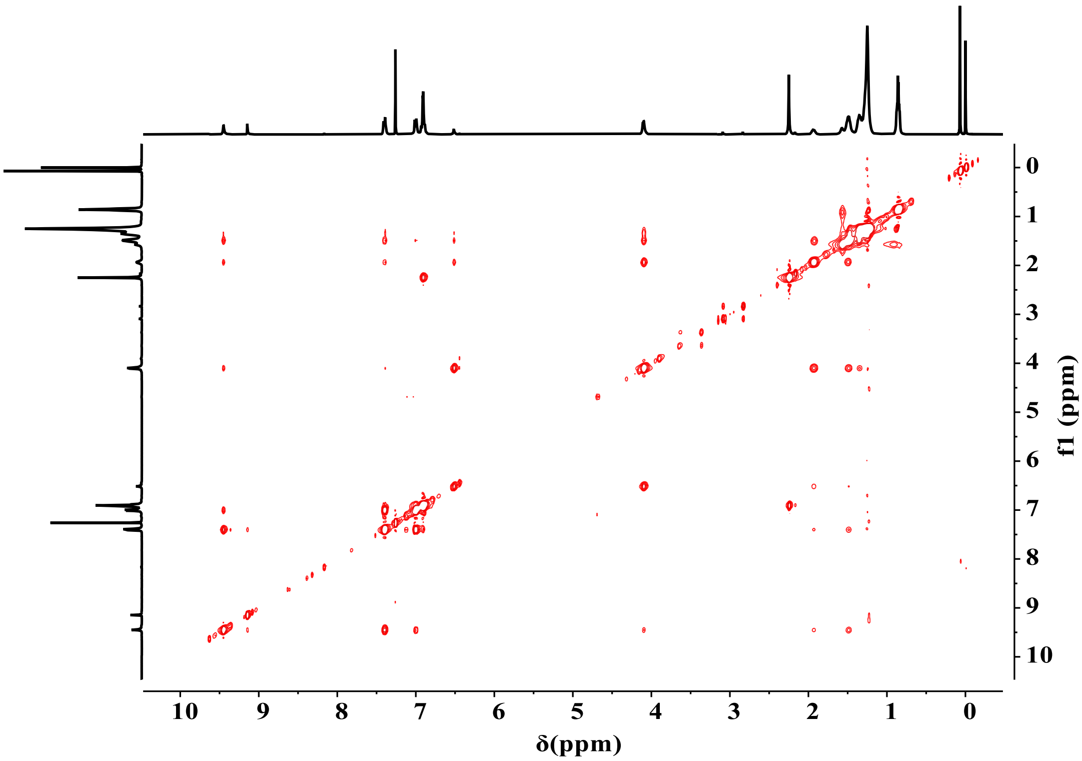

Figure S7. 2D-NOESY spectrum of compound **1a** (400 MHz, $CDCl_3$, 298 K, mixing time = 0.4s)

2. MALDI-TOF-MS Spectra

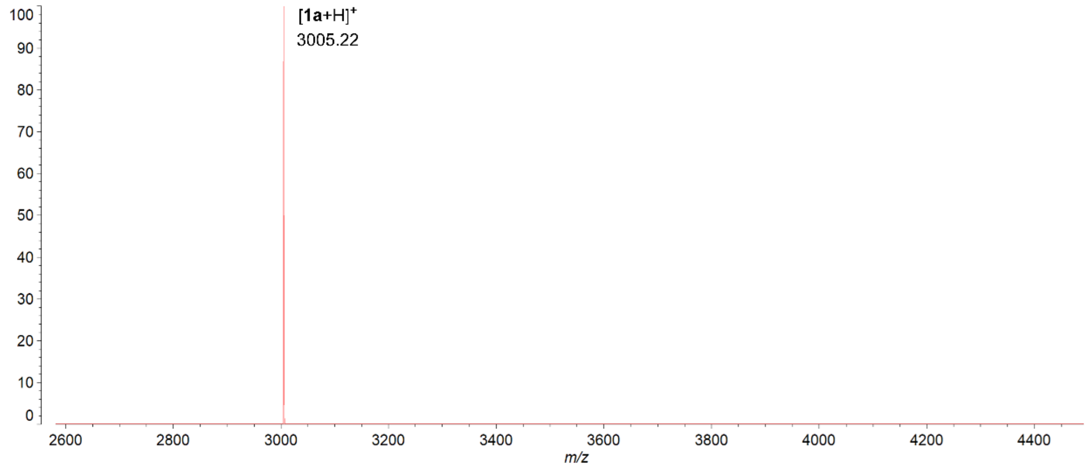

Figure S8. Partial MALDI-TOF-MS spectrum of compound **1a**

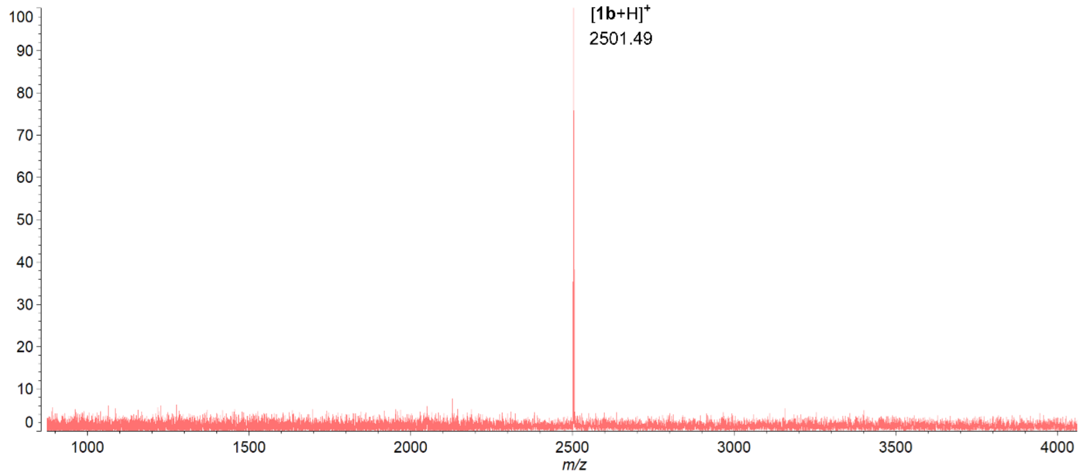

Figure S9. Partial MALDI-TOF-MS spectrum of compound **1b**

3. FT-IR Spectra

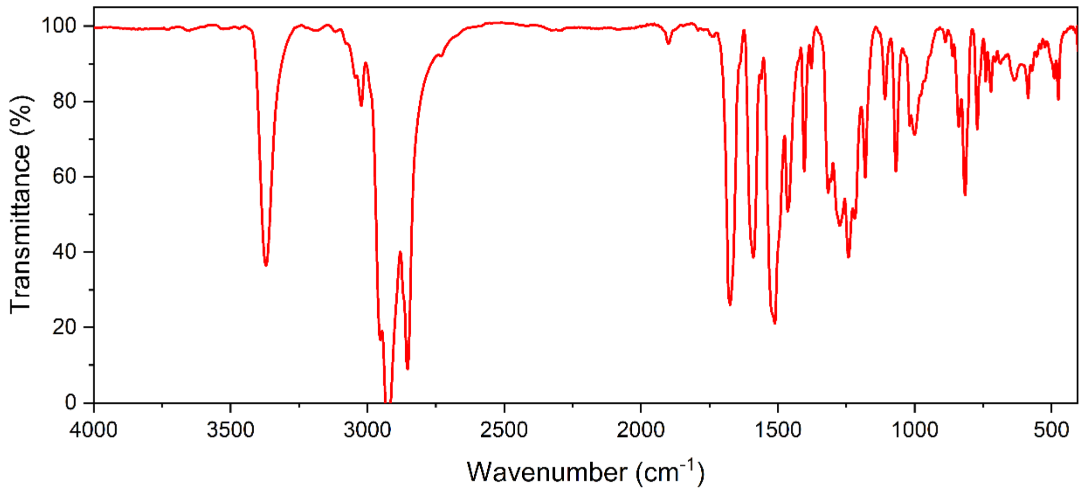

Figure S10. FT-IR spectrum of compound **1a**

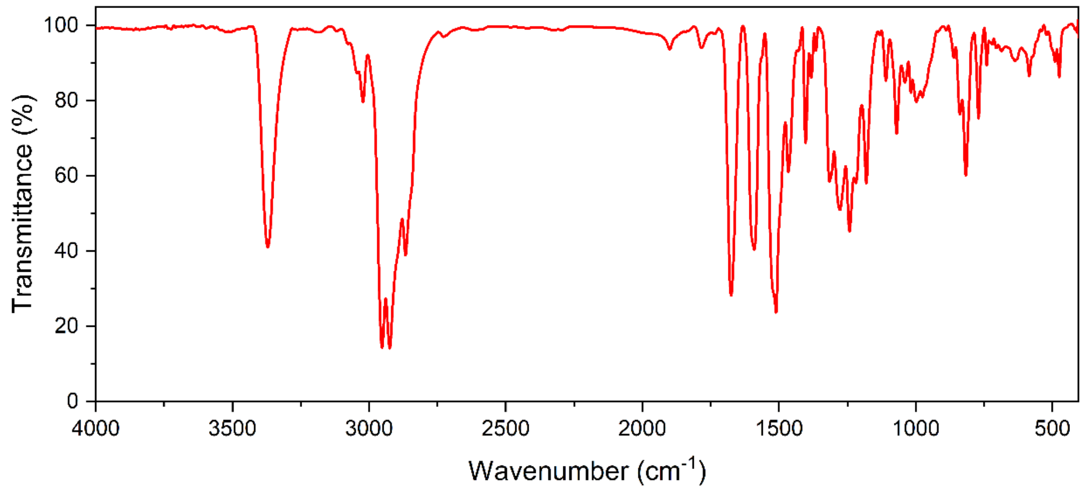

Figure S11. FT-IR spectrum of compound **1b**

4. Thermogravimetric Analysis

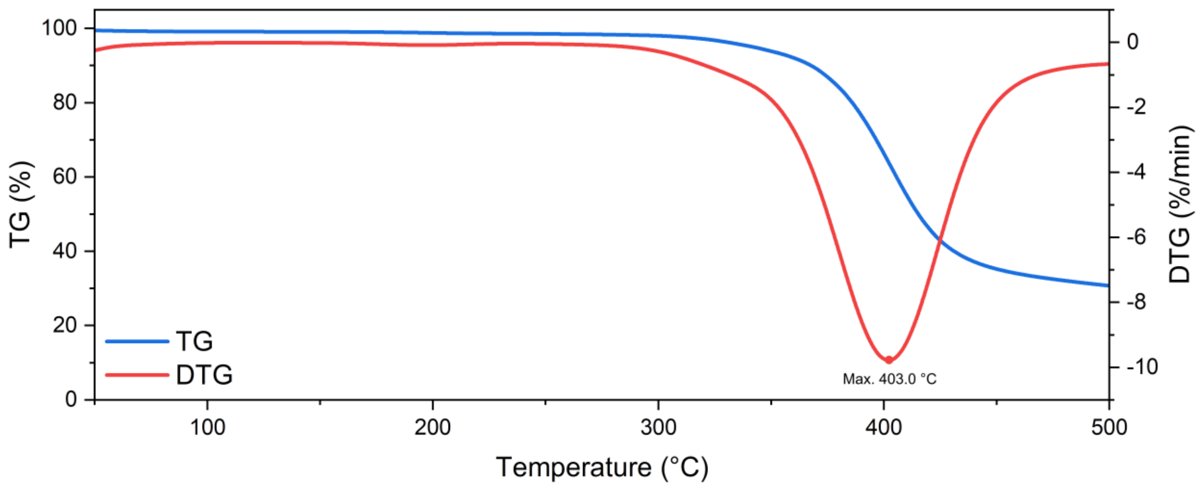

Figure S12. TGA and DTG diagrams of compound **1a** (under nitrogen atmosphere, heating rate = 10 °C/min)

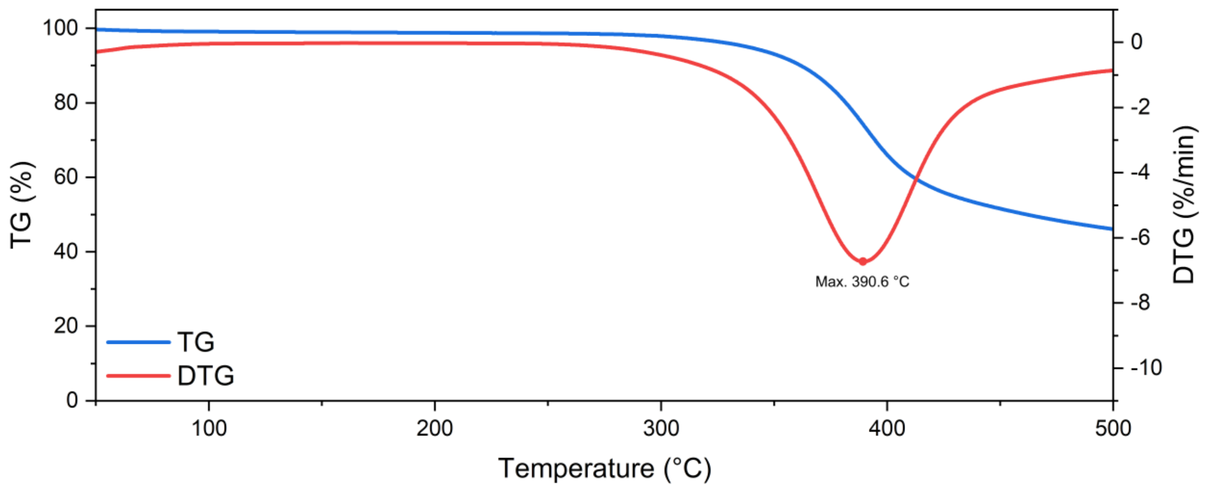

Figure S13. TGA and DTG diagrams of compound **1b** (under nitrogen atmosphere, heating rate = 10 °C/min)

5. Differential Scanning Calorimetry Results

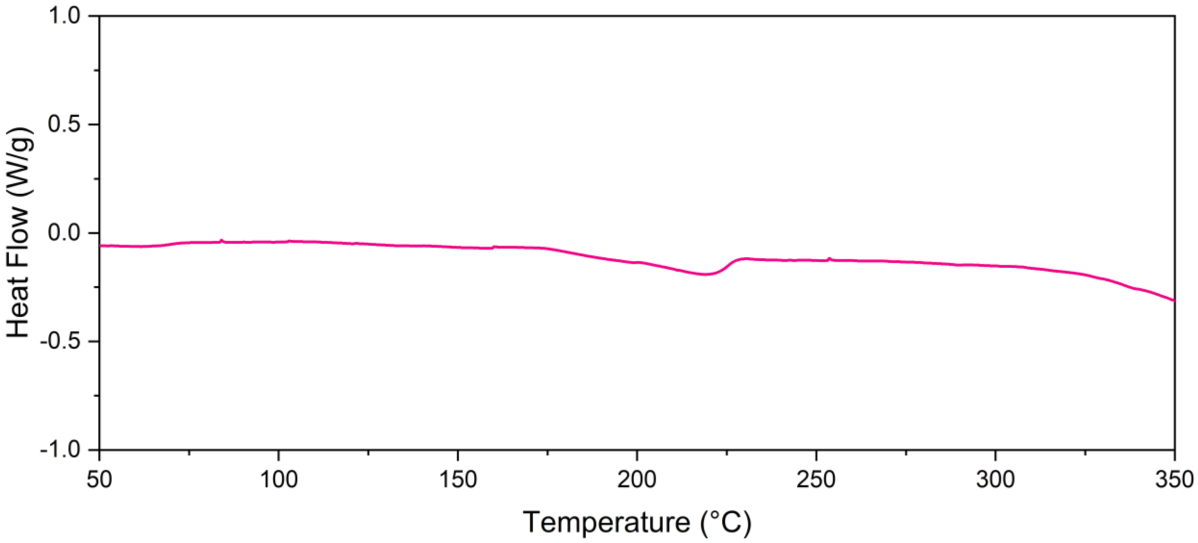

Figure S14. DSC diagrams of compound **1a** (under nitrogen atmosphere, heating rate = 10 °C/min)

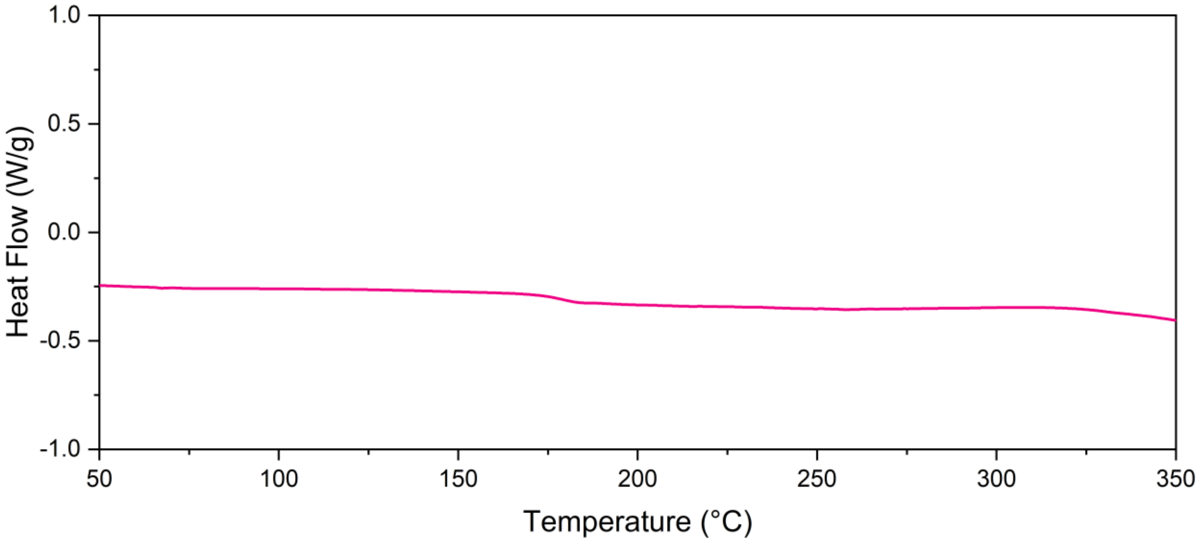

Figure S15. DSC diagrams of compound **1b** (under nitrogen atmosphere, heating rate = 10 °C/min)

6. Quantum Yield Results

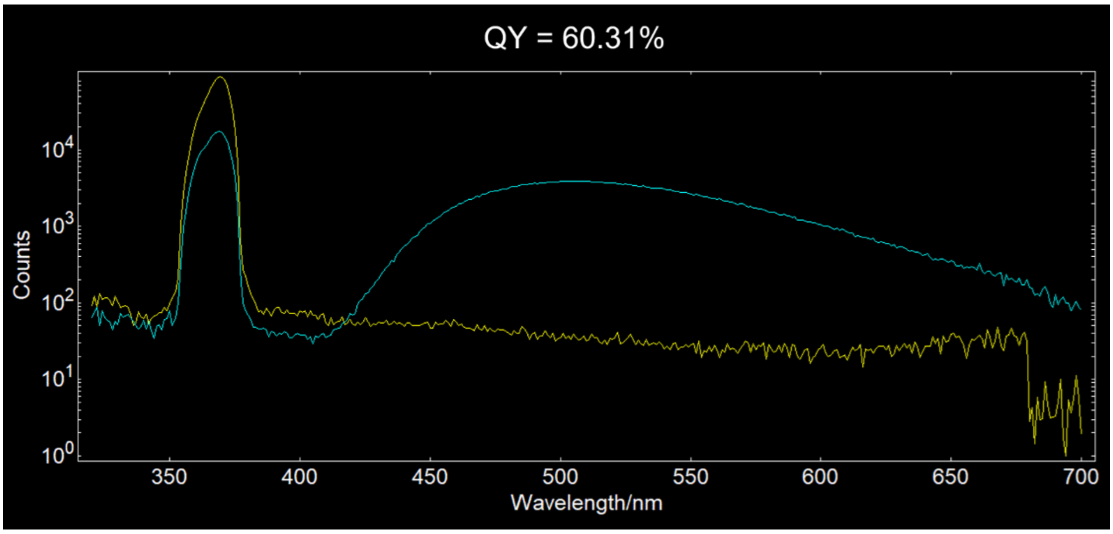

Figure S16. Quantum yield result of compound **1a** (Excitation range: 355 nm - 377 nm; luminescence range: 430 nm – 700 nm; yellow line = blank; cyan line = **1a**)

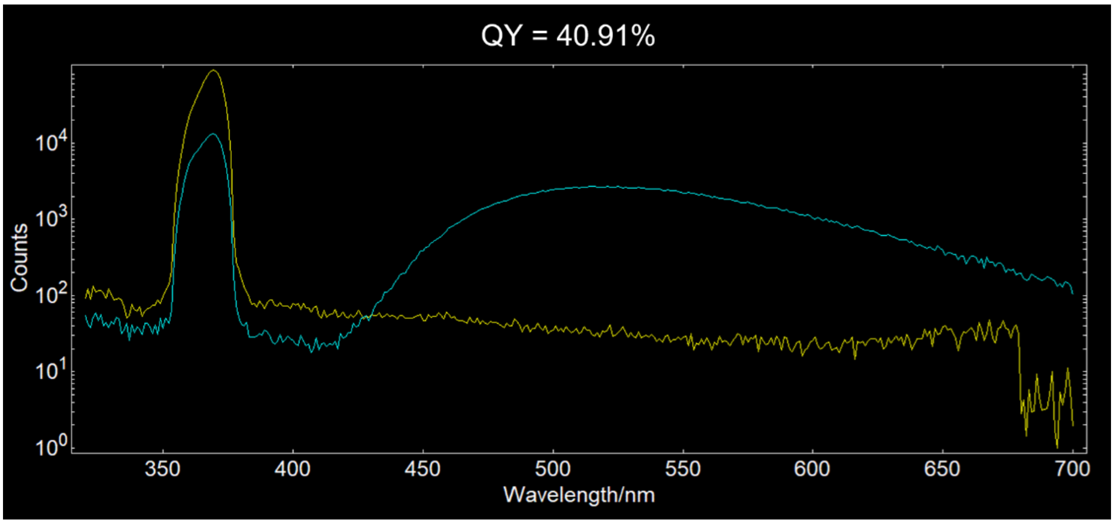

Figure S17. Quantum yield result of compound **1b** (Excitation range: 355 nm - 377 nm; luminescence range: 430 nm – 700 nm; yellow line = blank; cyan line = **1b**)

7. Transient Fluorescence Decay Spectra

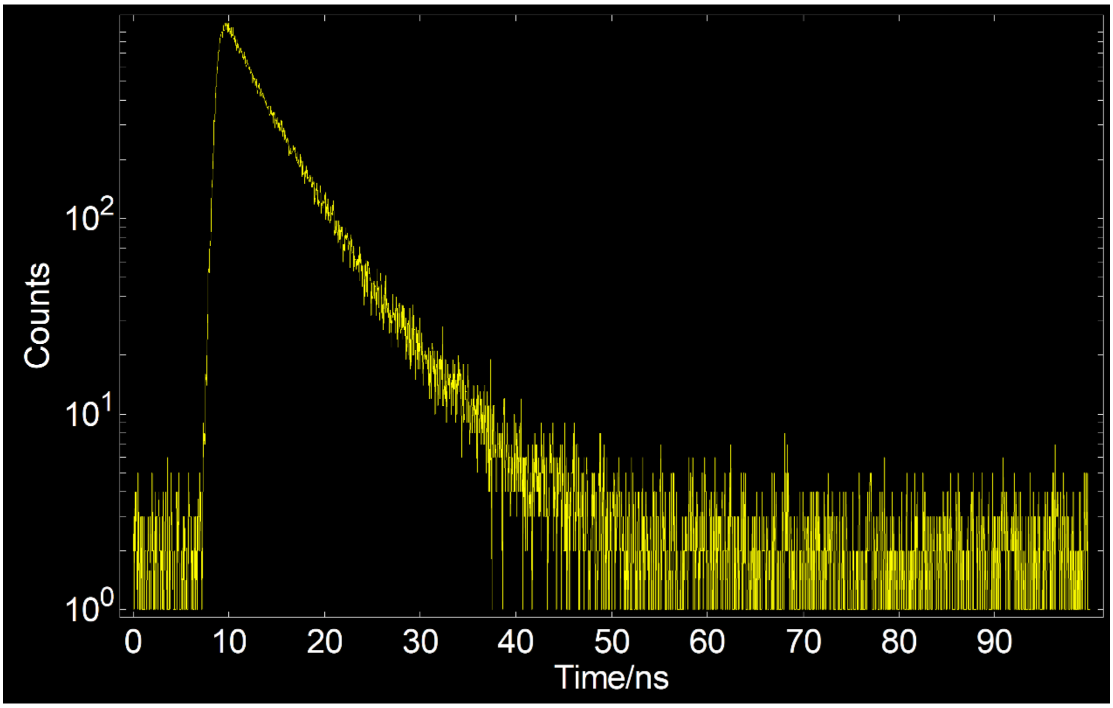

Figure S18. Transient fluorescence decay spectra of compound **1a** (298 K, Excitation wavelength = 340 nm, Emission wavelength = 500 nm)

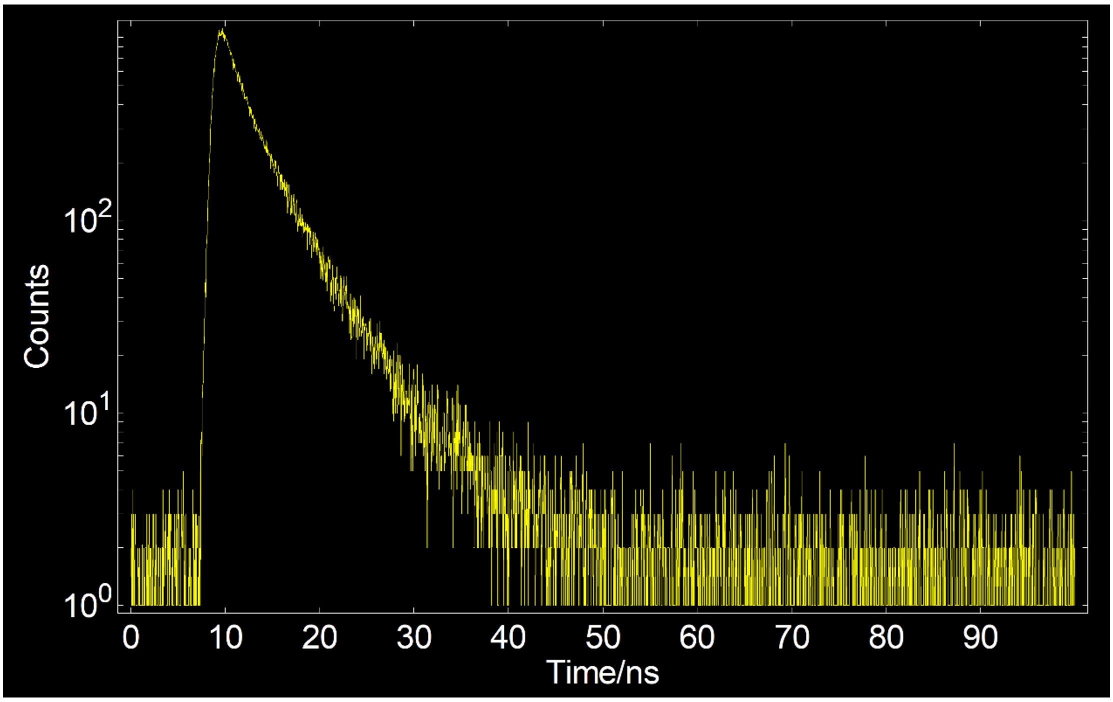

Figure S19. Transient fluorescence decay spectra of compound **1b** (298 K, Excitation wavelength = 340 nm, Emission wavelength = 500 nm)

8. DFT Calculation Result

Standard orientation for the optimized structure of macrocycle **1c.**

Atom	Coordinates (Angstroms)		
	X	Y	Z
C	12.59042	-3.99954	-1.97116
C	11.36269	-4.52377	-1.56996
C	11.23612	-5.23696	-0.36714
C	12.3988	-5.41967	0.404305
C	13.62022	-4.8855	0.004769
C	13.74153	-4.16423	-1.19205
C	15.07262	-3.59273	-1.62162
C	9.933731	-5.84037	0.045723
C	8.752999	-5.14189	0.02332
C	10.02018	-7.26586	0.483175
C	7.42282	-5.81646	0.087477
C	8.703122	-3.65277	-0.06751
C	9.570218	-2.84084	0.686099
C	9.472901	-1.45636	0.653998
C	8.491857	-0.8216	-0.12542
C	7.616619	-1.61249	-0.8854
C	7.730698	-2.99911	-0.84578
C	6.362397	-5.27621	0.837118
C	5.093166	-5.84732	0.850981
C	4.842166	-7.00138	0.092847
C	5.890291	-7.55764	-0.65881
C	7.14957	-6.97312	-0.66493
C	9.41116	-7.70556	1.671374
C	9.526951	-9.02709	2.093865
C	10.25689	-9.96684	1.35328
C	10.87088	-9.52975	0.172645
C	10.76634	-8.20584	-0.24883
C	10.40478	-11.3925	1.831035
N	8.451375	0.586307	-0.10339
C	7.386548	1.396981	-0.42217
H	9.281464	1.052541	0.237863
O	6.311855	0.968088	-0.82675
C	7.574802	2.87991	-0.20714
C	8.792751	3.591783	-0.17897
C	8.797265	4.986572	-0.06699

C	7.594923	5.693212	0.047513
C	6.362036	5.007493	0.080935
C	6.399589	3.619281	-0.0626
O	9.950688	2.869056	-0.27445
C	11.20151	3.544947	-0.3168
O	7.563524	7.058175	0.140205
C	4.990663	5.599564	0.304047
O	4.07444	4.889759	0.70336
N	4.836513	6.93293	0.002774
H	5.659823	7.411988	-0.33724
C	-2.87803	12.85578	-1.97248
C	-1.80053	12.0643	-1.58423
C	-1.08975	12.33317	-0.40152
C	-1.49634	13.44193	0.360862
C	-2.58215	14.2247	-0.02619
C	-3.29264	13.95056	-1.20174
C	-4.44311	14.8246	-1.64343
C	0.09265	11.51367	-0.00037
C	0.080056	10.14169	-0.00377
C	1.290262	12.30886	0.404718
C	1.330787	9.328905	0.052681
C	-1.18497	9.351548	-0.06334
C	-2.30717	9.701403	0.709372
C	-3.45728	8.923691	0.705566
C	-3.53018	7.750933	-0.06371
C	-2.4218	7.383036	-0.84174
C	-1.27811	8.17613	-0.83039
C	1.402311	8.147704	0.813079
C	2.532835	7.335882	0.823386
C	3.64957	7.69002	0.050871
C	3.597879	8.867896	-0.71238
C	2.460619	9.664198	-0.71538
C	1.993847	12.02238	1.587555
C	3.086807	12.79058	1.979448
C	3.52475	13.87874	1.212262
C	2.821196	14.17075	0.03686
C	1.720049	13.41153	-0.35376
C	4.692891	14.72735	1.657217
N	3.592069	-7.64866	0.046971
H	3.593545	-8.60115	-0.29334
C	2.361217	-7.11437	0.350265

O	2.206525	-5.96657	0.752135
C	1.160891	-8.00303	0.124767
C	-0.05915	-7.33852	-0.01294
C	-1.28831	-7.98301	-0.16344
C	-1.28316	-9.39404	-0.14711
C	-0.07867	-10.0981	-0.03979
C	1.135578	-9.41325	0.080786
O	2.331965	-10.0715	0.169973
C	2.35462	-11.4936	0.193719
O	-2.48873	-10.0332	-0.24912
C	-2.53131	-11.4539	-0.30623
C	-2.47573	-7.0738	-0.3743
O	-2.30486	-5.92233	-0.75853
N	-3.71415	-7.59484	-0.07842
H	-3.72957	-8.55168	0.248986
C	8.782413	7.790713	0.171888
C	-12.6229	-3.7872	2.027053
C	-11.4101	-4.33445	1.611245
C	-11.3114	-5.04928	0.406812
C	-12.4863	-5.20861	-0.35142
C	-13.6925	-4.65184	0.062837
C	-13.786	-3.92964	1.261615
C	-15.1009	-3.33359	1.70762
C	-10.0259	-5.6788	-0.01998
C	-8.83208	-5.00266	-0.01029
C	-10.1455	-7.10236	-0.45562
C	-7.51447	-5.69972	-0.08603
C	-8.75641	-3.51434	0.077356
C	-9.61047	-2.69081	-0.67834
C	-9.49063	-1.30798	-0.65048
C	-8.49914	-0.68712	0.126883
C	-7.63649	-1.48994	0.888809
C	-7.77303	-2.87468	0.853331
C	-6.44905	-5.17229	-0.83786
C	-5.18967	-5.76437	-0.86216
C	-4.95403	-6.92721	-0.11273
C	-6.00724	-7.47111	0.640806
C	-7.25656	-6.86582	0.657502
C	-9.5564	-7.55502	-1.64912
C	-9.70434	-8.8737	-2.07034
C	-10.4478	-9.79763	-1.32318

C	-11.0417	-9.34758	-0.13724
C	-10.905	-8.02622	0.283124
C	-10.6305	-11.2198	-1.79919
N	-8.43621	0.719855	0.1012
C	-7.35832	1.514775	0.415791
H	-9.25931	1.198268	-0.24014
O	-6.28925	1.070499	0.818669
C	-7.5254	3.000015	0.198845
C	-8.73315	3.729244	0.170676
C	-8.7177	5.123721	0.056206
C	-7.50551	5.812898	-0.0609
C	-6.28254	5.109583	-0.09394
C	-6.33986	3.722316	0.051966
O	-9.9012	3.023285	0.269037
C	-11.1425	3.716525	0.310786
O	-7.45481	7.177023	-0.15675
C	-4.90308	5.681772	-0.31911
N	-4.72882	7.012386	-0.01642
H	-5.54401	7.502964	0.326684
C	-8.66308	7.926791	-0.19099
O	-3.99779	4.958978	-0.71999
H	12.65513	-3.45717	-2.91215
H	10.48738	-4.38192	-2.19641
H	12.33772	-5.98474	1.330318
H	14.49791	-5.03563	0.630813
H	15.8414	-4.37233	-1.69403
H	15.00081	-3.10401	-2.59858
H	15.44064	-2.84821	-0.90369
H	10.3196	-3.30351	1.319523
H	10.14273	-0.85592	1.266563
H	6.847464	-1.14015	-1.47962
H	7.036509	-3.59145	-1.43391
H	6.530991	-4.37788	1.423185
H	4.293864	-5.39923	1.423743
H	5.704098	-8.43907	-1.26938
H	7.932527	-7.40997	-1.2758
H	8.839466	-6.99934	2.265573
H	9.044646	-9.33369	3.019955
H	11.443	-10.2362	-0.42559
H	11.26144	-7.89442	-1.16446
H	9.507498	-11.7343	2.358979

H	10.58848	-12.078	0.996598
H	11.24787	-11.4966	2.527794
H	9.735541	5.521527	-0.06903
H	5.458006	3.08274	-0.06069
H	11.95566	2.763771	-0.42291
H	11.25629	4.222315	-1.1773
H	11.38536	4.10641	0.607597
H	-3.40448	12.62112	-2.89559
H	-1.50178	11.22262	-2.20156
H	-0.95887	13.68557	1.273147
H	-2.88094	15.06649	0.595448
H	-5.2162	14.24224	-2.1572
H	-4.91086	15.33204	-0.79282
H	-4.10919	15.60364	-2.34248
H	-2.27009	10.58731	1.334447
H	-4.30071	9.208037	1.331794
H	-2.45688	6.475874	-1.42802
H	-0.42898	7.866954	-1.43221
H	0.546457	7.849077	1.410665
H	2.551359	6.425057	1.404685
H	4.447572	9.141783	-1.33479
H	2.439674	10.55387	-1.33582
H	1.677608	11.18489	2.201894
H	3.607093	12.54256	2.902541
H	3.137583	15.00815	-0.58201
H	1.189128	13.6688	-1.26618
H	5.448082	14.12926	2.179377
H	5.178612	15.21859	0.807155
H	4.3732	15.51827	2.349569
H	-0.05137	-6.25488	-0.00298
H	-0.08623	-11.1781	-0.05009
H	3.405018	-11.7703	0.295197
H	1.791657	-11.884	1.049793
H	1.954104	-11.9166	-0.73585
H	-3.5854	-11.7134	-0.41467
H	-1.97311	-11.8319	-1.17096
H	-2.13764	-11.9043	0.613373
H	8.494708	8.838156	0.273431
H	9.397934	7.49808	1.030839
H	9.354017	7.657402	-0.75489
H	-12.6662	-3.24415	2.968877

H	-10.525	-4.20994	2.227536
H	-12.4467	-5.77385	-1.2785
H	-14.5803	-4.78452	-0.5529
H	-15.884	-4.09825	1.785736
H	-15.0088	-2.84995	2.685388
H	-15.462	-2.57929	0.996414
H	-10.3676	-3.14336	-1.30994
H	-10.151	-0.69847	-1.2644
H	-6.85966	-1.02826	1.481412
H	-7.08804	-3.47669	1.442552
H	-6.60574	-4.26759	-1.4173
H	-4.38597	-5.32579	-1.43616
H	-5.8322	-8.35922	1.244957
H	-8.04315	-7.29331	1.270276
H	-8.97505	-6.86119	-2.24852
H	-9.23718	-9.19053	-3.00076
H	-11.6234	-10.0417	0.46618
H	-11.3852	-7.7042	1.202988
H	-9.74244	-11.5841	-2.32767
H	-10.8298	-11.8996	-0.96365
H	-11.4767	-11.3043	-2.49491
H	-9.64807	5.672276	0.058351
H	-5.4061	3.172232	0.050146
H	-11.9073	2.946024	0.418355
H	-11.1875	4.395817	1.170345
H	-11.3189	4.27921	-0.61433
H	-8.36037	8.969521	-0.29733
H	-9.28321	7.639204	-1.04833
H	-9.23615	7.805891	0.736621
